# Factors that influence implementation at scale of a community-based health promotion intervention for older adults

**DOI:** 10.1186/s12889-019-7984-6

**Published:** 2019-12-03

**Authors:** Joanie Sims-Gould, Heather A. McKay, Christa L. Hoy, Lindsay Nettlefold, Samantha M. Gray, Erica Y. Lau, Adrian Bauman

**Affiliations:** 1 Centre for Hip Health and Mobility, University of British Columbia and Vancouver Coastal Health Research Institute, 7F-2635 Laurel Street, Vancouver, British Columbia V5Z 1M9 Canada; 2 School of Public Health, University of Sydney, Sydney, New South Wales Australia

**Keywords:** Physical activity, Public health, Seniors, Implementation science

## Abstract

**Background:**

Despite the many known benefits of physical activity (PA), relatively few older adults are active on a regular basis. Older adult PA interventions delivered in controlled settings showed promising results. However, to achieve population level health impact, programs must be effectively scaled-up, and few interventions have achieved this. To effectively scale-up it is essential to identify contextual factors that facilitate or impede implementation at scale. Our aim is to describe factors that influence implementation at scale of a health promotion intervention for older adults (Choose to Move). This implementation evaluation complements our previously published study that assessed the impact of Choose to Move on older adult health indicators.

**Methods:**

To describe factors that influenced implementation our evaluation targeted five distinct levels across a socioecological continuum. Four members of our project team conducted semi-structured interviews by telephone with 1) leaders of delivery partner organizations (*n* = 13) 2) recreation managers (*n* = 6), recreation coordinators (*n* = 27), activity coaches (*n* = 36) and participants (*n* = 42) [August 2015 – April 2017]. Interviews were audio-recorded and professionally transcribed and data were analyzed using framework analysis.

**Results:**

Partners agreed on the timeliness and need for scaled-up evidence-based health promotion programs for older adults. Choose to Move aligned with organizational priorities, visions and strategic directions and was deemed easy to deliver, flexible and adaptable. Partners also noted the critical role played by our project team as the support unit. However, partners noted availability of financial resources as a potential barrier to sustainability.

**Conclusions:**

Even relatively simple evidence-based interventions can be challenging to scale-up and sustain. To ensure successful implementation it is essential to align with multilevel socioecological perspectives and assess the vast array of contextual factors that are at the core of better understanding successful implementation.

## Background

*“Most good ideas, however, do not spread with such ease. They require the backing and energies of committed individuals and organizations to design and carry out strategies for expansion that are carefully tailored to the realities of their settings.”* [1]

In Canada and the United States, the population of older adults (> 65 years) is projected to double over the next 25 years [2, 3]. The most rapidly growing segment of North America’s population are those over age 85 [3, 4]. Indeed, longevity is an unprecedented societal achievement. However, living longer will inevitably generate substantial health care costs unless governments invest in innovative health promotion strategies that support and sustain health across what possibly comprises three decades of older adult life.

Despite the many known health benefits of physical activity (PA) [5], older adults are the least active citizens in Canada [6]. Regular PA effectively ameliorates the risk of chronic disease [7], decreases the odds of functional limitation, including ‘mobility-disability’ [8] and social disengagement [9], substantially. These broad-sweeping physical and social health benefits are crucial to maintain older adult independence. Strategies that integrate PA into opportunities for older adults to socially engage, may diminish risk of chronic disease, preserve older adults’ mobility and independence [7], and mitigate the increasing number of lonely and socially isolated older adults [10, 11]. During the past five decades the focus of PA research has broadened from an almost exclusive focus on fitness to include different choice-based domains of PA for health that span walking for transportation to household activities [12] and those that increase opportunities to socially connect [13].

Although there are promising results from older adult PA interventions delivered in controlled settings [14], to achieve population level health impact programs must be effectively scaled-up. To do so, interventions are designed with scalability in mind [15], and implementation is guided by implementation or scale-up frameworks [16]. Ultimately, multi-levels and multi-sectors [17, 18], collaborative partnerships, ongoing stakeholder interactions [19] and defined progression through stages of implementation [20] are key to scale-up and sustained implementation [21]. However, to our knowledge, only eight studies targeting older adults and PA were scaled-up (defined as “the process by which efficacious health interventions are expanded under real world conditions into broader policy or practice” [22, 23]), with mixed outcomes [24–31]. Thus, there is an implementation – scale-up gap within health promotion that warrants our attention. Specifically, we need to better understand 1) how an intervention that is effective at smaller scale can be adapted to meet the needs of diverse settings and in different older adult populations and 2) how interventions can be adapted for delivery at large scale to enhance health of older adults.

We aim to fill this gap in the literature by sharing our approach to the implementation and phased scale up of a health promotion intervention called Choose to Move (CTM) for older adults in British Columbia, Canada. We previously published the conceptual frameworks for implementation and scale up that underpin our research approach [32], the translational formative evaluation that informed design and implementation of the intervention at scale up [33], and the positive impact of CTM on older adults’ health during the first two phases of scale up [13]. Thus, our objective is to describe factors that influence successful implementation of CTM during Phase 1 and 2 (2016–2017) scale up. Results will be used to adapt CTM for broader scale-up in Phase 3.

## Methods

In this section, we provide a brief overview of; 1) the CTM intervention; 2) context for implementation and scale-up of CTM, 3) the implementation framework that guides CTM implementation, and 4) the role of partner organizations. Using qualitative methods, we focus on factors that influenced implementation of CTM across four levels of delivery partners and from the perspective of older adult participants. All study procedures were approved by the Clinical Research Ethics Board at the University of British Columbia [H15–02522] and the Research Ethics Board at Simon Fraser University [22,015 s0614]. All participants provided informed written consent prior to each interview. There was no financial incentive for participating in the interview.

### The intervention – choose to move

CTM is a 6-month, choice-based, flexible, scalable, health promotion intervention for low active (< 150 min of moderate to vigorous PA/wk) older adults (60+ years). To guide CTM content and delivery, we adopted core elements of the effective Community Healthy Activities Model Program for Seniors (CHAMPS; choice-based, telephone assisted approach). CHAMPS was delivered at scale by 13 diverse community agencies [24, 34–36]. Increased PA participation was of similar magnitude as reported for efficacy studies – despite greater diversity in participants’ ethnicity, socio-economic status, and health conditions [27, 37]. We then conducted a translational formative evaluation in order to assess context for implementation, adapt where needed, and pilot our approach and measurement tools [33].

**Fig. 1:**
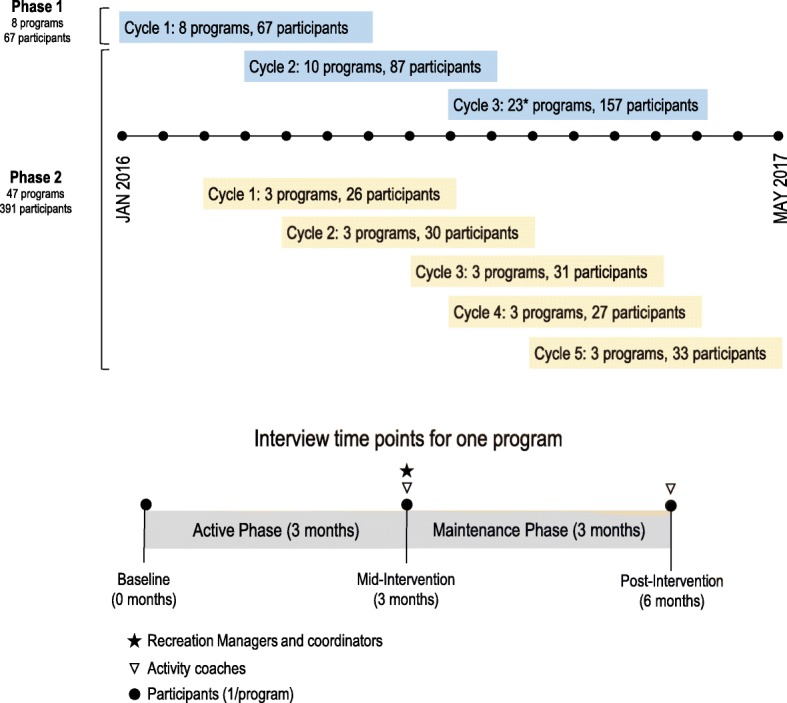
Phased delivery of Choose to Move and timing of interviews by participant group. Blue and yellow bars represent programs delivered by different delivery organizations. Leaders of delivery organizations were interviewed before (or during) phase 1 and at the end of phase 2. Timing of interviews for other groups (Recreation Managers, Recreation Coordinators, Activity Coaches and Participants) are illustrated in the bottom portion of the figure. All Recreation Managers at sites delivering 3 or more programs were interviewed once at the mid point of the final program. All Recreation coordinators were interviewed at program mid-point the first and last time their site delivered a program. All Activity coaches were interviewed at program mid-point the first and last time they delivered a program; a subset were interviewed post-intervention as well. Participants were interviewed at baseline, mid-point and post-intervention. *Although 23 programs were initiated during this cycle, 1 program was cancelled after baseline measures were collected. Adapted with permission from “Implementation of a co-designed physical activity program for older adults: positive impact when delivered at scale,” by McKay H, Nettlefold L, Bauman A, Hoy C, Gray SM, Lau E, and Sims-Gould J, 2018, BMC public health, 18 [1]:1289. CC BY 4.0

CTM is currently being delivered in three phases at increasingly greater scale across the province of British Columbia (BC) (Fig. 1). Phase 1 [2016; *n* = 8 programs, *n* = 67 participants] and Phase 2 [2016–2017; *n* = 47 programs, *n* = 391 participants] comprised implementation of CTM at smaller scale, in urban, suburban and small urban communities. Phase 3 [2018–2020] targets broad scale-up [13]. In this study we focus on implementation of CTM during Phases 1 and 2 (*n* = 55 programs, *n* = 458 participants). In total, CTM was delivered at 26 unique sites with either 1 (*n* = 15 sites), 2 (n = 8 sites), 3 (n = 3 sites) or 5 (n = 3) program cycles delivered during phases 1 and 2.

CTM is comprised of three core elements delivered by trained activity coaches. First, a 60-min one-on-one consultation with activity coaches assists participants to set and maintain PA goals that cater to their physical capacity and preferences (create an Action Plan). Second, participants attend four 60-min Motivational Group Meetings (once/month in months 1–2; twice in month 3 to connect socially with other participants (max 12/group) and with their activity coach. Third, activity coaches call participants regularly by phone (15 min/call on average) to monitor progress, address challenges, and modify the Action Plan as needed (three times in month 1; twice/month in months 2 and 3; once/month in months 4–6). As CTM is supported through a government grant, there is no cost to participants. We use multiple implementation strategies [38] with our partners to deliver CTM (Table 1).

**Table 1:** Choose to Move (CTM) Implementation Strategies

CTM Implementation Strategies [38]	Descriptions
Conduct needs assessment (at provincial partner level)	Prior to CTM implementation, the CTM project team (project team)* conducted formative evaluation using semi-structured focus interviews to assess i) Older adults’ acceptability of CTM ii) Delivery partners’ perceived adaptability of CTM to context and population iii) Activity coaches’ perceived feasibility of implementing CTM implementation of the intervention by Activity Coaches (to assess feasibility) and to identify and pilot evaluation tools and methods to assess effectiveness of CTM at scale. We collaborated with both delivery partners to adapt the program to their organizational context
Develop community partnership and obtain formal commitments	The project team partnered with the two community organizations to deliver CTM at their affiliated facilities. Both organizations have signed contract agreement committing on program delivery.
Develop program materials and tools	The project team developed and provided the following materials for the delivery organization. These include materials for: i) Program managers • Recruitment materials • Descriptions of program coordinators and activity coaches hiring process and job descriptions ii) Program coordinators • Outline of implementation and evaluation tasks iii) Activity coaches • Presentation materials on health topics for motivational group meeting sessions • Tools to record participants attendance and responses during one-on-one Action Planning and telephone check-ins.
Centralized technical assistance	The project team functioned as the prevention support system to provide centralized technical assistance to the program coordinators, managers and activity coaches.
Conduct dynamic training	The project team provided a 1-day training for activity coaches. Training content included overview of CTM, motivational interviewing techniques, active listening skills. They were provided with skills demonstration, opportunities to practice the learned skills and ask questions.
Provide on-going consultation	The project team provided on-going telephone consultations to each delivery sites throughout the intervention period. The purpose of these phone calls was to identify and troubleshoot implementation issues. These included: • Regular phone call (weekly to start, then monthly as Phase II progressed) with provincial coordinators • On-going email communications to provide additional support
Use advisory boards and workgroups	During CTM implementation, the project team formed two advisory committees that provide ongoing feedback we use to adapt the program as needed throughout the intervention period. Both advisory committees meet annually. • The Community Advisory Committee comprises older adult participants, recreation coordinators, and activity coaches from partner organizations and members of the Active Aging Research (AART) team. This committee shares lessons learned during the implementation of CTM. • The Leadership Advisory Committee comprises leaders of delivery partner organizations and members of AART. This committee was the organizational lens we used to monitor the implementation of CTM in collaboration with partner organizations and to assess the need for further adaptation of CTM to meet the specific needs and capacity of delivery organizations before scale-up. Both advisory committees meet annually.
Stage implementation scale up	CTM was first piloted in DP2 (8 communities) in Phase I before a larger scale roll out in DP1 and DP2 in Phase II (48 communities). This pilot provided opportunity for delivery partners to provide feedback on the feasibility of CTM implementation and identify barriers and facilitators of implementation. This feedback was then used to refine the intervention and implementation plan in Phase II.

* The Choose to Move project team (project team) was convened by the Active Aging Research Team to support delivery of CTM. Specifically, the project team comprised of two principal investigators (HM, JSG), international research collaborators, a program manager and several research assistants to support day-to-day operation and program evaluation

Reproduced with permission from “Implementation of a co-designed physical activity program for older adults: positive impact when delivered at scale,” by McKay H, Nettlefold L, Bauman A, Hoy C, Gray SM, Lau E, and Sims-Gould J, 2018, BMC public health, 18 (1):1289. CC BY 4.0

### Context for implementation and scale-up

In response to the escalating rates of chronic disease [39], physical inactivity, and social isolation in Canada [6, 40, 41], BC Ministry of Health developed Active People, Active Places, a PA Action Plan for BC [42] .Older adults were identified as a priority area. A funding partnership was established between the Ministry of Health and the Active Aging Research Team (AART; www.activeagingrt.ca).

### Implementation framework

**Fig. 2:**
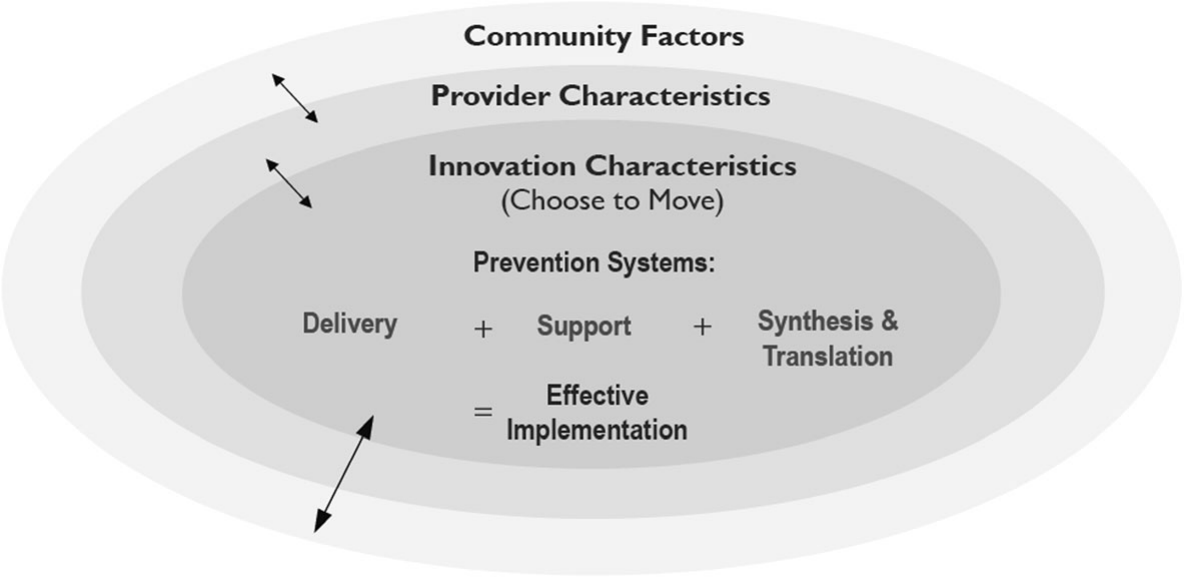
The framework for effective implementation. Implementation of the Choose to Move is guided by the prevention delivery system and its organizational capacity, the prevention support system and the prevention synthesis & translation system. These critical components are embedded within a larger context of provider characteristics and community factors (outer rings). Interactions between all components in the outer and central rings are illustrated by bidirectional arrows. Reprinted with permission from McKay HA, Sims-Gould J, Nettlefold L, Hoy CL, Bauman AE. Implementing and Evaluating an Older Adult Physical Activity Model at Scale: Framework for Action. Translational Journal of the ACSM. 2017;2 [2]:10–9. https://journals.lww.com/acsm-tj/pages/default.aspx. Figure originally adapted from American Journal of Community Psychology, Volume 41 [3, 4], Dupre, J.A. and Durlak E.P., Implementation Matters: A Review of Research on the Influence of Implementation on Program Outcomes and the Factors Affecting Implementation, 327–350, Copyright (2008), with permission from John Wiley and Sons

We provided details of the conceptual frameworks for implementation and evaluation, guiding principles, the intervention, and evaluation methods that guide our work at length in a previous publication [32]. Briefly, the Framework for Effective Implementation [43] guided development, implementation and evaluation of CTM (Fig. 2). This framework highlight six categories of contextual factors that influence effective implementation; 1) the innovation (CTM), 2) the prevention delivery system (e.g., activity coaches, recreation center coordinators/ managers and delivery partner organizational leads), 3) the prevention support system (e.g., our Active Aging Research Team), and 4) the prevention synthesis and translation (research) system (e.g., our Active Aging Research Team) - all nested within the socioecologic context of 5) provider and 6) community characteristics [43, 44]. These different ‘levels of influence’ also guide our evaluation approach.

Although we aligned our approach with one of more than 60 published frameworks [45], there are many common elements among them. These often include, attributes of the intervention, factors that support implementation, delivery strategy, characteristics of the adopting community, the broader socio-political context, and the use of research and evaluation to inform the scale-up process [46]. However, others contend that there is no single or straightforward implementation framework that offers a formula for success [21]. As implementation research evolves it will, “help implementers to better understand the complex array of contextual factors, such as politics, socio-cultural norms and beliefs, and the fiscal environment, that can influence scale-up success” [29].

### Delivery partners and the support unit

Consistent with the Framework for Effective Implementation [43] community partners served along a continuum from strategy (leaders of delivery partner organizations), to operations (recreation coordinators and managers), to on the ground delivery (activity coaches) – together they comprised the *delivery system*. Two major delivery partner organizations delivered 56 CTM programs between January 2016 and May 2017 in 26 community centres or other facilities. They were selected based on their established relationships with recreation centres, their capacity to coordinate delivery of programs at scale, and their desire to sustain implementation of a health promotion intervention for older adults across BC, in future. We defined this operationally as CTM being aligned with the vision and mission of the delivery partner organization.

We convened a project team (from AART) that served as CTM implementation *support and research systems* [43, 44]. The support unit provided leadership, training, and created a communications plan and governance structure. The support unit engaged delivery partners to design, implement, and evaluate CTM to meet the needs of older adults. The support unit ensured that implementation strategies varied as little as possible across sites.

### CTM implementation evaluation

We consider implementation evaluation an essential component of intervention studies as 1) it provides context to interpret participant level health outcomes [13], 2) it can be adopted by researchers who wish to evaluate scale-up of most health focused interventions, and 3) implementation outcomes can be used to guide *adaptation* of an intervention to setting and population, as a means to enhance likelihood of successful scale-up and sustainability, in the future. We aligned our implementation evaluation with the Framework for Effective Implementation [43]. We present our results across levels of influence (from leaders of delivery partner organizations to CTM participants) as per the socioecologic model [43]. We identified these stakeholders using the influence approach reported by Colvin [47].

Four members of the *support and research system* conducted semi-structured interviews by telephone with all participant groups. The interview questions were tailored to participant group and focused on perceived levels of support, challenges and opportunities, adaptations, and perceived fit of CTM to the local context. Interviews used an interview guide, specific to each participant group and time point. Guided by our implementation framework, the project team developed questions for the interview guide. Questions for the activity coach and participant interview guides were used previously in our translational formative evaluation [33]. Questions for the recreation manager and coordinator interview guides were tested in those groups before Phase 1. For all groups, we interviewed the same individual across time points. Table 2 provides examples of questions we asked to identify factors influencing implementation. Interviews were audio-recorded and professionally transcribed. Interviewers were trained by the lead author to take high level notes to serve as back up should the audio recording be faulty or unclear.

**Table 2:** Five participant groups across levels of influence with sample questions for each group

Participant Group	Sample Questions
Leaders of delivery partner organizations	How does offering this type of program fit with the strategic direction of your organization moving forward? What needs to be in place for this type of programming to be successful in your organization on a longer term? Across the province?
Recreation managers	What needs to be in place for a program like CTM to be successful in your centre on a longer term? What is the likelihood that your organization will continue to deliver this type of programming as part of usual practice?
Recreation coordinators	What factors have helped your organization implement CTM? What has made it challenging for your organization to implement CTM?
Activity coaches	Generally, how well did the implementation of CTM proceed for you? Did you modify any of the program components? If so, why?
Participants	What are your favourite parts of the program?

#### Leaders of delivery partner organizations

We interviewed leaders of delivery partner organizations before or during Phase 1 (August 2015–September 2016; *n* = 13) and again at the end of Phase 2 (March–April 2017; *n* = 6). Interviews were designed to ascertain the political and organizational climate surrounding PA promotion (context for delivery), the fit of CTM with organizational priorities, perceived (baseline) and actual (follow-up) facilitators and barriers to delivering CTM across BC, and willingness to continue to deliver CTM (follow-up only; sustainability). Interviews were conducted by one trained interviewer for Phase 1 and another at follow-up and lasted approximately 12 min (range = 5 to 22 min).

#### Recreation managers and recreation coordinators

We interviewed recreation managers (n = 6) once (at program mid-point) at six sites where programs were delivered in 3 or more cycles. We interviewed recreation coordinators at program mid-point the first and last time their site delivered a program during Phases 1 or 2 (April 2016 – February 2017; *n* = 27 completed one interview; *n* = 14 completed two interviews). We asked recreation managers and coordinators about facilitators and barriers to program delivery, suggestions for improvement, any adaptations to the implementation process, and willingness to continue delivering CTM. One trained interviewer conducted all interviews that lasted approximately 18 min (range = 7 to 46 min). Due to small sample sizes, we aggregate recreation manager and recreation coordinator data together to preserve anonymity.

#### Activity coaches

We interviewed all activity coaches at program mid-point, the first (*n* = 23) and last (*n* = 13) time they delivered CTM during Phase 1 and 2 [March 2016 – January 2017]. We interviewed a subset (randomly selected) of activity coaches (*n* = 19) at 6-months. We asked similar questions at both 3-month and 6-month interviews, including facilitators and barriers to CTM delivery, adaptation made to the intervention or its implementation, suggestions for improvement, lessons learned, and success stories. If they delivered programs during five cycles (n = 1), we interviewed activity coaches at 3-months of Cycle 1, 3 and 5. One trained interviewer conducted all interviews. Interviews lasted approximately 27 min (range = 10 to 54 min).

#### Participants

We randomly selected one participant per site from those who consented to the interview portion of the study so as to ensure geographic diversity. Our CTM participants are mostly white (85%) women (77%); this is also reflected in the subset of participants completing the interview (91% white, 63% women). We interviewed these participants at baseline (*n* = 42), 3-months (*n* = 38) and 6-months (*n* = 34) of their program [January 2016 – April 2017]. Two trained interviewers conducted all interviews. Interviews lasted approximately 11 min (range = 3 to 25 min).

### Analysis

Interview recordings were transcribed verbatim by a professional transcriptionist. Data were de-identified and imported to NVivo 11 (QSR International, 2015) for data analysis. For all participant groups, interview data were analyzed using framework analysis [48–50], an appropriate analytic approach for qualitative studies with specific questions, a pre-designed sample, and a-priori issues [50]. In framework analysis, data are sifted, charted and sorted in accordance with key issues and themes using five steps: 1) familiarize; 2) identify a thematic framework; 3) index; 4) chart; and 5) map and interpret [50, 51]. First, two team members read through a subset of transcripts to establish a sense of the interviews (familiarize). Through team meetings we developed a preliminary thematic framework, consisting of themes and sub-themes, based on key issues and commonalities emerging from the transcripts (identify). Using Nvivo 11 software, one team member coded interviews based on the thematic framework, with discussion among other team members, as new codes and sub-themes were identified (index). Full paragraphs were coded so that contextual meaning was not lost. Data were then summarized by charting illustrative quotes that best exemplified the themes (chart). As part of the interpretive process a series of team meetings (*n* = 6) were held to discuss the data for common themes and sub-themes (map and interpret). We used a number of strategies to reinforce the rigor of our analysis: crosschecking full transcripts against original audio files for quality and completeness and reflexive memoing throughout the data generation and data analysis process [52].

## Results

**Fig. 3:**
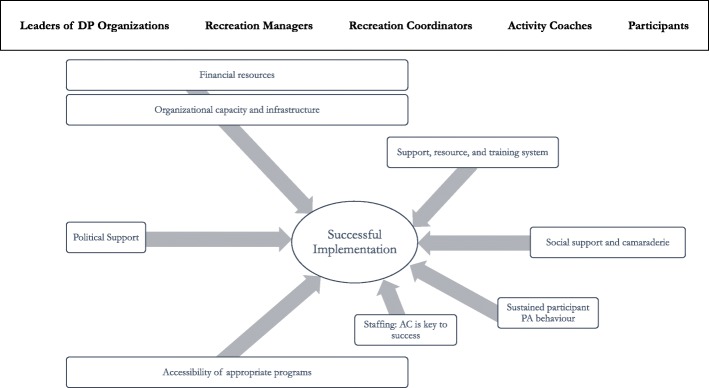
Themes emerging from our analysis. We illustrate key themes that emerged from our analysis. Some themes emerged across multiple levels; others were only identified by one participant group

The *support and research system* (comprised of an AART project team) worked in close proximity with CTM delivery partners across levels. The central role of the *support and research system* was to provide ongoing oversight across levels of delivery partners with a focus on implementation. Importantly, the *support and research system* monitored intentional or unintentional adaptations to the intervention and its implementation. We describe key elements that delivery partners felt either supported or hindered implementation and scale-up of CTM, below. The main themes are illustrated in Fig. 3.

### Leaders of delivery partner organizations

Leaders of delivery partner organizations discussed the organizational context [43] for program delivery. They felt that while there is some political support for PA in the province, there are very few (if any) systemically organized programs for older adults. Against this backdrop they highlighted the challenge for older adults to become active or to access programs:

*“So there’s definitely a need for that population to be physically active. There’s definitely a need and the demand and market for older adult fitness is for sure there. I think with the growing aging population this demand is needed--not going anywhere. It’s just a matter of, you know, accessibility … ”*

Further, this group noted that programmatic offerings vary by region, and it is often challenging for older adults to navigate the community-based system in order to find suitable programs. Overall, CTM was considered timely, necessary, relevant, and scalable. This speaks to the importance of the organizational context for delivery. Leaders noted complete alignment with their organizational priorities, vision, and strategic directions. As noted by one leader:

*“this gives us the resources to actually fulfill our own mandate, and our own mandate as an organization is to enable communities in their attempts to build their communities and build strong, healthy resilient individuals.”*

Leaders indicated that their organizations have the infrastructure and capacity but not the financial resources to offer CTM. By virtue of its choice-based nature, CTM was deemed flexible and scalable. In this way, leaders highlighted their organization’s capacity to sustain CTM: *“And we do have the infrastructure, so we have the staff who can take it on and provide training and the passion”*. On the other hand, leaders expressed concern about how CTM might be sustained in the absence of ongoing provincial funding. They saw currently intact linkages with Ministry of Health as essential for scale-up of CTM and a possible mechanism to ensure sustainability. CTM was considered a good place to refer older adults who are currently engaged with BC’s health care system (i.e. awaiting treatment or involved with home and community care) and where PA was not contraindicated. There was also discussion about the need to reach older adults who are more isolated and/or marginalized.

“*I would say one of the issues is just how do you reach the people that need the program, you know, because just given the fact that the people who need the program often may be isolated, well, how do you reach them?”*

When speaking specifically about design of CTM, leaders noted that the social aspects of the program were paramount to older adults. They identified the need to provide opportunities for older adults to socialize and that CTM created those opportunities. Leaders also highlighted that an evidence-based program developed by researchers made CTM more ‘credible’ for those delivering CTM and for older adults enrolled in CTM.

### Recreation managers/coordinators

Recreation managers/coordinators were very positive about CTM. One manager stated “*it is a beautiful model*” and “*a good fit with the mandate of our organization*”. The social components were deemed an excellent component of CTM. Recreation managers/coordinators also noted the essential need for external funding to run the program and to find the right staff to deliver CTM; highlighting questions around sustainability. Sustainability was deemed uncertain without long term financial commitments to CTM. Specifically, they highlighted the need to recover costs to support activity coaches.

*“And for us we don’t have a budget to subsidize programs. So all our programs have to be cost-recovery, so in order for us to keep this on-- as part of our regular practice we would need to cover the cost for our instructors and/or space.”*

They highlighted that charging a fee for CTM might be one way to do this, although that could pose a potential barrier to participation among older adults.

Through implementation of CTM, managers/coordinators gained valuable insight into time management, communications, and recruitment needs of CTM. Central to delivery of CTM, the pivotal role of the activity coach surfaced time and again. Recreation managers/coordinators expressed concern about the lack of older adult certified instructors in certain communities, issues with staff turnover, and challenges related to bringing instructors in from outside a recreation centre as this may lead to unfamiliarity with processes specific to that city/centre. They noted that it is much easier when an activity coach is hired who is already part of a specific centre’s programming. Finally, they emphasized that it was very helpful when activity coaches took ownership of CTM and in so doing, assisted with recruitment, registration, and screening. This sentiment also speaks to limited organizational capacity.

*“I think one of the big ones is that the Choose to Move (activity coach) is somebody we have a relationship with already. It’s a staff member at the facility so it’s just-- I find that that’s-- that’s helped with the implementation of the program”*

They noted that materials provided for CTM participants were appropriate and contributed to CTM being “easy to implement”. The role of the *support and research system* was viewed as instrumental to successful implementation of CTM.

*“the support we receive from (the support and research system) is really important. Certainly, the training materials, the weekly, bi-weekly check-ins that we have with (support and research system lead) are also very important to us. It gives us an opportunity to touch base, kind of just provide updates but also kind of problem solve any challenges that we may be experiencing as well.”*

They also stated how crucial it was to ensure adequate lead time to allow recreation centres to promote CTM, to generate other communication materials (e.g. recreation calendars), and to provide information sessions for all those involved in recruitment and delivery of CTM (e.g. front desk staff). This would ensure that those delivering CTM at every level had a clear understanding of CTM and could adequately answer any questions they were asked. This, in turn, was thought to increase recruitment efforts of more vulnerable or marginalized older adults (i.e. those who may not normally participate in recreation centre activities). Finally, they deemed centralized training and opportunities for activity coaches to engage with one another as highly beneficial.

*“Yeah, so when we first started there was-- the timing of it didn’t allow us to get it into our normal kind of Fit Living guide cycle. So we felt like we had to do a, you know, ton of extra work to spread the word, otherwise it wouldn’t be out there.”*

### Activity coaches

Activity coaches highlighted that CTM was an ‘excellent’ program, well laid out, and easy to deliver. They also noted that the *support and research system* was central to CTM’s success. Activity coaches felt their role was very rewarding; there was a strong desire for CTM to continue. When speaking about other rewarding aspects of CTM, the significant changes in participants’ level of activity and connectedness were noted.

*“I mean, success stories to me is just everybody that’s still moving. I mean, people that I called on their six month and they’re-- they still, you know, want to brag to me about, you know, what they’re doing and how they’re feeling. Or if they’ve fallen off the wagon and, you know, push the restart button and got going again, how they felt when they were off the wagon. And how they couldn’t believe it, right. I just felt so sorry and I didn’t feel good and this and that. It’s like yeah. Great. So what did you do about it? I started exercising again. Awesome! Good for you. Good choice and you made it without me.”*

In addition to changes in activity and connectedness, improvements in participants’ health status was highlighted as important. Activity coaches noted that they made a number of ‘small tweaks’ to the intervention to facilitate implementation. For example, some activity coaches modified group meeting content due to time constraints and/or relevance to the community context. Other activity coaches modified the active travel component due to community built environment characteristics that limited opportunities for active travel. In addition, activity coaches used face-to-face meetings or email as an alternative mode for check-ins (original mode was telephone) due to participant preference or to accommodate participant schedules. Finally, activity coaches distributed supplemental resources based on participant requests for further information on a topic of interest.

### Participants

Participants were pleased with and very much enjoyed participating in CTM. They agreed overwhelmingly that activity coaches were central to their continued participation and key to the success of CTM. Specifically, participants noted how positive attitudes and encouragement offered by activity coaches made a tremendous difference.

*“He’s extremely patient with everyone. He’s very supportive. I think when he was on the phone there was never-- I never felt like I was being rushed off the phone. It was always, you know, what can I do to help? Is there anything more I can help you with?”*

Most participants felt that the number of check-in calls from their activity coach was adequate. After the first three months of CTM, they felt that PA started to become a part of their everyday routine. Their activity coaches’ words of support and guidance resonated in their minds. Testament to them enjoying CTM, participants wished it ran for a longer duration. They also offered suggestions as to how best to foster more social opportunities amongst participants. Related to this, participants noted that group meetings provided important opportunities for participants to share their experiences, discuss PA related issues, and generally interact with others.

*“It’s just nice to hear what other people are doing, though. It’s nice to hear how, you know, they have been motivated. And I think it encourages everybody when you, you know, hear somebody else say, well, this is what happened with me. And so, yeah, that part-- I like the sharing part.”*

Participants cited an array of health benefits from their participation in CTM. The three most often noted benefits were improvements in physical health, an increase in knowledge and awareness about the importance of PA, and an increase in motivation to engage in PA. For health benefits, joint health most commonly improved. The most common barriers to participating in CTM were unrelated to implementation factors. They most often related to individual health conditions such as injury, recent surgery, illness, or weather.

## Discussion

The World Health Organization [53] and many countries and their regions world-wide voiced the need to develop and communicate the need for effective strategies to implement health promoting PA interventions at scale. However, few jurisdictions succeed in doing so [22, 54]. Of evidence-based health related programs and practices, about 65% of attempts to scale-up fail [55, 56]. This is due in large part to the prescriptive and sometimes complex nature of these interventions and the varied and dynamic contexts in which they are delivered. There has been a global call for researchers to design, implement, and conduct implementation evaluations to unravel factors that influence implementation of PA interventions at scale [57, 58].

We responded to this call and extend the existing literature by identifying factors across five levels of influence, that facilitate or impede implementation and scale-up of a health promotion intervention for older adults. We also capture the perspective of those whom the intervention is designed to positively benefit – older adults. Our approach aligns with multilevel socioecological perspectives and implementation frameworks that acknowledge the vast array of contextual factors at the core of successful implementation [43, 44, 59]. Within scale up, Yamey [21] suggests that four elements are crucial for successful implementation; an innovation (intervention) that is simple and technically sound, strong leadership and active engagement by a range of stakeholders (delivery partners), the use of a wide variety of strategies to implement the innovation, and a robust evaluation (research). We heard from stakeholders that they considered these elements a core part of CTM.

First, all delivery partners expressed that CTM *fit well* with provincial organization and local facility mandates and that it was simple and evidence-based. There was also strong consensus that CTM was appropriate and effective for older adults. This was directly attributed to CTM’s concrete, yet flexible design and choice-based nature. This reflects the extensive (2-year) process we adopted to co-design CTM with community partners. It also reflects our commitment to a continuous cycle of feedback (from partners across levels) and adaptation as per the Knowledge-to-Action Cycle [60]. Responding to the needs of delivery partners and adapting to community context was supported by our phased approach to implementation [13, 32] from formative evaluation to small scale-up [Phase 1; 2016] to implementation at medium-scale [Phase 2; 2016–2017] in preparation for implementation at large scale [Phase 3; 2018–2020]. The notion of scaling-up in a phased manner to capitalize on lessons learned along the way has been cited as important in the expansion of other health-based initiatives [61, 62]. Older adults also identified many elements that enticed them to engage in CTM. Of note, they found the ‘choice-based’ concept that is core to CTM relatively easy to understand and most appreciated having choice rather than a prescribed program of exercise.

Second, the capacity for CTM to be adapted speaks to engaging a range partners at the outset to co-design the intervention and its implementation [63], and to program flexibility and scalability. Strong leadership across levels of partners facilitated implementation. The *support and research system* also provided strong leadership and was seen as the backbone for implementation and for guiding ongoing adaptation of CTM. Leadership has been noted elsewhere as a key element that promotes implementation success [21].

Third, we used multiple implementation strategies with our partners to implement CTM; our interview questions were designed to provide participants an opportunity to comment on them. For example, at the operational level recreation managers/coordinators identified three major challenges to implementation. In smaller communities, it was difficult to find and retain trained fitness leaders and to recruit participants. Recreation coordinators also underestimated the time and effort required to fill and administer programs in many communities. We address these issues as we implement CTM during Phase 3.

Activity coaches were the front facing deliverers of CTM and in this role were the major point of contact for older adult participants. Activity coaches employed a number of strategies to recruit and retain participants. Many activity coaches had long standing connections to their communities and used these linkages to draw older adults into CTM. Participants spoke at length about the importance of a knowledgeable activity coach who responded to their individual needs and concerns.

Finally, we developed and implemented CTM with a strong focus on evaluation. The important role of the project team from AART as *support and research system* emerged as key to successful implementation and evaluation of CTM across delivery partners. This reflects the *support and research system*’s ability to integrate evaluation activities and engender commitment across levels of influence to achieve positive benefits for older adults [43, 44, 64]. Many implementation frameworks note the crucial nature of central support and knowledge synthesis and translation for effective implementation [1, 44, 56]. The *support and research system* built capacity within community organizations by providing specialized training, through a formal communications plan enacted within and across levels, and through continual feedback about the intervention and the evaluation. Two-way communications also provided delivery partners the opportunity to provide the *support and research system* with key evaluation data and to communicate about program adaptations to CTM that they felt would better support its implementation. We agree that a comprehensive implementation evaluation can be used “to organize and promote synthesis of research findings … which will further stimulate theory development” [56]. We envision that our implementation evaluation will serve as one template to evaluate health promotion interventions, and in so doing contribute to filling the implementation-scale-up gap. We also note, an important next step is to design a study with a more specific focus on fidelity to implementation strategies.

We would be remiss to ignore the impact of financial resources on successful implementation of CTM. Funding for CTM reflects a commitment to enhance the health of older adults as one current priority within BC Ministry of Health. We secured competitive grant funding to conduct a comprehensive evaluation. Although there is no permanent, long term commitment from government to fund CTM, as we continue to rigorously evaluate scale up (including impact, implementation and cost-utility) we become better positioned to advocate for CTM’s sustained scale up and delivery over the longer term.

## Conclusion

Even relatively simple evidence-based interventions can be a challenge to scale-up and sustain [57, 58], as they are often implemented within complex geographical, sociocultural and ethnically diverse environments. Older adults, whose longevity now spans three decades or more, represent a wide range of physical and cognitive capacity. Further, the environments where they live may or may not include access to support networks, community resources and/or health promotion programs that can be adapted to their needs and capacity. If we are to shift the trajectory away from increased chronic disease, mobility-disability, and problems such as loneliness – new strategies that engage stakeholders across multiple levels with a view to implementation at scale are needed. Health promotion models like CTM may be one solution. CTM underscores the importance of a simple, technically sound intervention, committed and integrated partners, a wide array of implementation strategies, and a comprehensive implementation evaluation that provides ongoing feedback so that implementation strategies can be customized to context. CTM illustrates one approach to implementation and scale-up of an effective health promotion intervention that can be adapted by policy makers, academics, and service deliverers.

## Data Availability

The datasets generated for this study are not publicly available due to the restrictions of the ethics approval.

## Abbreviations

AART: Active Aging Research Team
BC: British Columbia
CTM: Choose to Move
PA: Physical activity
